# Translating radiology reports into plain language using ChatGPT and GPT-4 with prompt learning: results, limitations, and potential

**DOI:** 10.1186/s42492-023-00136-5

**Published:** 2023-05-18

**Authors:** Qing Lyu, Josh Tan, Michael E. Zapadka, Janardhana Ponnatapura, Chuang Niu, Kyle J. Myers, Ge Wang, Christopher T. Whitlow

**Affiliations:** 1 Department of Radiology, Wake Forest University School of Medicine, Winston-Salem, NC 27103 United States; 2 Biomedical Imaging Center, Rensselaer Polytechnic Institute, Troy, NY 12180 United States; 3 Puente Solutions LLC, Phoenix, AZ 85065 United States

**Keywords:** Artificial intelligence, Large language model, ChatGPT, Radiology report, Patient education

## Abstract

The large language model called ChatGPT has drawn extensively attention because of its human-like expression and reasoning abilities. In this study, we investigate the feasibility of using ChatGPT in experiments on translating radiology reports into plain language for patients and healthcare providers so that they are educated for improved healthcare. Radiology reports from 62 low-dose chest computed tomography lung cancer screening scans and 76 brain magnetic resonance imaging metastases screening scans were collected in the first half of February for this study. According to the evaluation by radiologists, ChatGPT can successfully translate radiology reports into plain language with an average score of 4.27 in the five-point system with 0.08 places of information missing and 0.07 places of misinformation. In terms of the suggestions provided by ChatGPT, they are generally relevant such as keeping following-up with doctors and closely monitoring any symptoms, and for about 37% of 138 cases in total ChatGPT offers specific suggestions based on findings in the report. ChatGPT also presents some randomness in its responses with occasionally over-simplified or neglected information, which can be mitigated using a more detailed prompt. Furthermore, ChatGPT results are compared with a newly released large model GPT-4, showing that GPT-4 can significantly improve the quality of translated reports. Our results show that it is feasible to utilize large language models in clinical education, and further efforts are needed to address limitations and maximize their potential.

## Introduction

Since being released by OpenAI in November 2022, ChatGPT, a state-of-the-art natural language processing (NLP) model, has received global attention and over 100 million users owing to its human-like expression and reasoning abilities [1, 2]. ChatGPT answers users’ general queries as if it were a human and can perform various tasks, including poem composition, essay writing, and coding including debugging. Compared with previous NLP models such as BERT [3], XLNet [4], and generative pre-trained transformer (GPT) [5], ChatGPT is a quantum leap characterized by several characteristic features, including a larger model with more parameters, chain of thought prompting, and training with reinforcement learning from human feedback (RLHF). ChatGPT was developed based on GPT-3, which has 175 billion parameters; the aforementioned models have fewer than 200 million parameters. Prompted learning is used to effectively induce the reasoning process; RLHF injects high-quality human knowledge, helping align the results of ChatGPT to ensure it is friendly and safe for society [6].

Given the huge success of ChatGPT, studies have been conducted recently on adapting it for downstream tasks, such as writing a systematic literature review [7], medical school education [8], language translation [9], scholarly content generation for publication [9], and solving mathematical problems [10]. The clinical use of ChatGPT has also been extensively studied. Patel et al. [10] attempted to use ChatGPT to write patient discharge summaries and discussed their concerns. Biswas explored the use of ChatGPT for medical writing applications, including patient-care-related writing, medical publications, medical administrative documentation, and meeting summarization [11]. Jeblick et al. [12] investigated the quality of ChatGPT’s radiology report simplification and concluded that simplified reports were factually correct, complete, and not harmful to patients. Rao et al. [13] demonstrated the feasibility of using ChatGPT as an adjunct to radiology decision-making. Sarraju et al. [14] used ChatGPT to provide cardiovascular disease prevention recommendations and found that over 80% of ChatGPT responses were appropriate.

Radiology reports summarize expert opinions on medical images acquired using radiography, computed tomography (CT), magnetic resonance imaging (MRI), as well as nuclear, ultrasound, and optical imaging methods. The findings in these reports are instrumental in diagnosis and treatment. However, the medical terminology in such reports is often difficult to understand for patients without any medical background. With ChatGPT, it is now possible to re-express a professional report in plain language so that patients know the meaning of their radiology reports, which is invaluable in reducing anxiety, promoting compliance, and improving outcomes.

This study focuses on the performance of ChatGPT in translating radiology reports into layman versions. ChatGPT is asked to provide suggestions for both patients and healthcare providers based on the content of each radiology report; the quality of the provided suggestions were subsequently evaluated. Furthermore, the results of ChatGPT were compared with those obtained using the newly released GPT-4.

## Methods

### Report acquisition

To demonstrate the performance of ChatGPT on a set of representative radiology reports, 62 chest CT and 76 brain MRI screening reports were collected from the Atrium Health Wake Forest Baptist clinical database. All reports were generated between February $1^{st}$ and $13^{th}$. All reports were de-identified by removing sensitive patient information.

Chest CT screening reports followed the low-dose chest CT lung cancer screening protocol without contrast agents. The patients were between 53 and 80 years of age, with an average of 66.9 years (32 male, 30 female). The reports were finalized by 11 experienced radiologists and had an average of $278 \pm 57$ words. The reports were classified into six classes based on the overall Lung-RADS categories shown in each report (1, 1S, 2, 2S, 3, 4A). The statistics concerning chest CT screening reports are shown in Table 1.

**Table 1:** Statistics concerning chest CT screening reports

	1	1S	2	2S	3	4A	Overall
Category count	15	2	35	5	1	4	62
Category percentage	24%	3%	56%	8%	2%	6%	100%
Age (year)	65.2 ± 6.2	62.5 ± 6.4	67.3 ± 6.0	71.4 ± 5.6	60.0 ± 0	69.0 ± 8.8	66.9 ± 6.3

Brain MRI screening reports followed the brain tumor protocol with and without the use of a contrast agent. The patients’ ages ranged from 5 to 98 years, with an average of 55.0 years (45 male, 31 female). The reports were finalized by 14 experienced radiologists and had $247 \pm 92$ words. The reports were further classified into three classes based on the findings of metastases: no metastases, stable condition without newly emerging or growing metastases, and worsening condition with growing or newly emerging metastases, as shown in Table 2.

**Table 2:** Statistics concerning brain MRI screening reports

	No metastase	Stable	Worsening	Overall
Category count	11	40	25	76
Percentage	14%	53%	33%	100%
Age (year)	63.6 ± 11.5	47.9 ± 21.0	60.9 ± 16.8	54.5 ± 19.7

### Experimental design

In the experiments, ChatGPT was provided the following three prompts and recorded its responses:

- Please translate a radiology report into plain language that is easy to understand.
- Please provide some suggestions for the patient.
- Please provide some suggestions for the healthcare provider.

All the ChatGPT responses were collected in mid-February of 2023.

### Performance evaluation

After collecting all ChatGPT responses, two experienced radiologists (with 21 and 8 years of experience) evaluated the quality of the ChatGPT responses.

During report translation, the evaluation effort focused on three aspects: overall score, completeness, and correctness. The radiologists recorded the number of places with missing information and with incorrect information in each of the translated reports and assigned an overall score based on the 5-point system (1 for worst and 5 for best). A statistical analysis on the radiologists’ feedback was subsequently conducted; for example, if there were ten translated reports and radiologists found one place of missing information among them, it was concluded that there was an average of 0.1 places of information missing.

For suggestion evaluation, statistical analyses were performed to record high-frequency suggestions, the percentage of specific suggestions on a certain finding in the report, and the percentage of inappropriate suggestions that were not related to any finding in the report.

## Results

### ChatGPT-translated reports vs the original reports

Tables  3 and  4 show a comparison of the word counts between the original and translated radiology reports. Compared with the original radiology reports, ChatGPT generated plain language versions which were typically less verbose in both chest CT and brain MRI cases. For the chest CT reports, 85.5% of the translated results (53 of 62) were shorter than the corresponding original reports, with an overall length reduction of 26.7%. Specifically, ChatGPT reduced the length of the original reports by 20.5%, 29.0%, 29.0%, 54.0%, and 29.4% for reports belonging to the Lung-RADS categories of 1, 2, 2S, 3, and 4A respectively. The only exception was the 1S category, with a length increment of 13.3% after ChatGPT translation. In brain MRI radiology reports, 72.4% of the translated results (55 of 76) contained fewer words than the corresponding original reports, with an overall length reduction of 21.1%. Except for the “no mats” category with a slightly increase in words (1.8%), the reports in all the other categories were shorter after ChatGPT translation. Specifically, the plain language versions of reports in ‘stable’ and ‘worsening’ categories were 13.1% and 34.1% shorter than the original versions, respectively.

In a typical scenario, the paragraph was shortened when the radiology report stated that there was no abnormality several times; ChatGPT summarized all these negative findings in a single sentence. For example, if a chest CT report indicated, “PLEURA: No pleural thickening or effusion. No pneumothorax. HEART: Heart size normal. No pericardial effusion. CORONARY ARTERY CALCIFICATION: None. MEDIASTINUM/HILUM/AXILLA: No adenopathy,” ChatGPT translated the text into “The pleura, heart and blood vessels are normal, and there is no sign of cancer in the lymph nodes.”

In addition to shortening paragraphs and distilling information, the translated reports were patient-friendly and easier to understand by replacing medical jargon with common words. For example, if a chest CT report regarding the findings in lungs stated *“Granuloma seen in the right middle lobe 1 mm.”* ChatGPT translated the text into the following sentence: *“There is a small 1 mm area in the right middle lobe that looks like a granuloma, which is a small area of inflammation that is usually not concerning.”* The ChatGPT translation explained the medical terminology of granuloma along with its severity.

Another significant characteristic of translated reports is information integration. ChatGPT is capable of integrating information shown in different sections of the original report so that patients can better understand the report. A good example is a chest CT report. This report was compared with the scan conducted on August 6, 2021 in the comparison section. In the findings section, there was a sentence *“There is a right lower lobe granuloma 6 mm unchanged.”*. ChatGPT integrated the information shown in the comparison and findings sections, and generated the following sentence: *“There is also a 6 mm granuloma in the right lower lobe, but it has not changed since a previous CT scan done in August 2021.”*

**Table 3:** Comparison of the chest CT screening reports and their ChatGPT translations

	1	1S	2	2S	3	4A	Overall
Reports words	240.1 ± 45.7	243.5 ± 2.1	291.6 ± 56.4	316.4 ± 44.3	338.0 ± 0	298.3 ± 56.5	280.8 ± 56.9
Translation words	190.9 ± 43.4	276.0 ± 17.0	206.9 ± 49.0	224.4 ± 111.6	155.0 ± 0	210.5 ± 33.6	205.8 ± 53.9

**Table 4:** Comparison of the brain MRI screening reports and their ChatGPT translations

	No metastases	Stable	Worsening	Overall
Reports words	158.8 ± 17.5	228.1 ± 49.1	344.9 ± 83.5	256.5 ± 89.2
Translation words	161.7 ± 31.4	198.3 ± 44.7	227.2 ± 44.4	202.5 ± 47.5

### Evaluation of ChatGPT translations by radiologists

Two radiologists were invited to evaluate the quality of the translated reports. The evaluation was based on three metrics: number of places with information loss, number of places with misinterpreted information, and overall score. The overall score was based on a 5-point system in which a score of 5 indicates the best quality, whereas a score of 1 indicates the worst quality.

Table 5 lists the statistics on the radiologists’ evaluation results. ChatGPT is shown to perform well on both chest CT and brain MRI scan reports. There were only 0.097 places of missing information and 0.032 places of incorrect information on average per chest CT report, which corresponds to one in every 10.3 and one in every 31.3 translated reports, respectively. Among all translated chest CT reports, 76% of the results rated had an overall score of 5. Regarding brain MRI scan report translations, 5% of the results had missing information, with an average of 0.066 places of missing information per report. Meanwhile, 9% of translated reports contained incorrect information with an average of 0.092 places of incorrect information per report; 37% and 32% of all brain MRI scan results were rated with overall scores of 4 and 5, respectively. Overall, the average number of instances of missing and incorrect information among all results were 0.080 and 0.065, respectively, with a frequency of approximately once in every 12.5 and 15.4 reports, respectively. The average overall score of all results was 4.268, of which 27% and 52% were rated with overall scores of 4 and 5, respectively.

**Table 5:** Radiologists’ evaluation results

	Information missing	Incorrect information	Overall score
Chest CT	0.097	0.032	4.645
Brain MRI	0.066	0.092	3.961
Overall	0.080	0.065	4.268

### Evaluation of ChatGPT-generated suggestions

When providing suggestions to both patients and healthcare providers, ChatGPT claimed that it could not provide medical advice on treatment at the moment; however, it could provide general suggestions for patients and healthcare providers. A statistical analysis was conducted on the ChatGPT-provided suggestions. According to Tables 6 and 7, suggestions for patients and healthcare providers are highly relevant. For example, among the suggestions based on chest CT reports, the most frequently given suggestions for patients and healthcare providers include “follow up with doctors” and “communicate the findings clearly to patient,” respectively. In approximately 37% of all cases, ChatGPT provided specific suggestions based on the findings in the radiology report. For instance, there was a brain MRI report which noted an observation of paranasal sinus disease in the patient. It was stated in the report that “Paranasal sinuses: Air-fluid levels within maxillary sinuses.” ChatGPT provided the following suggestions to the patient and healthcare provider respectively: “Manage sinus symptoms: The report notes that there is air-fluid in the patient’s maxillary sinuses (paranasal sinus disease). The patient may want to discuss with their healthcare provider about how to manage any symptoms related to this” and “Evaluate sinus symptoms: The report notes the presence of air-fluid in the patient’s maxillary sinuses (paranasal sinus disease). As such, it may be appropriate to evaluate the patient for any symptoms related to this and determine if any treatment or management is necessary.”

**Table 6:** General suggestions based on chest CT reports

Suggestion for a patient	Frequency
Follow-up with doctors	100%
Follow-up with recommended appointments	100%
Quit smoking	98%
Maintain a healthy lifestyle	92%
Suggestion for a healthcare provider	Frequency
Communicate the findings clearly to the patient	100%
Schedule follow-up appointments	100%
Encourage smoking cessation	98%
Encourage a healthy lifestyle	65%
Consider referral to a specialist	40%
Monitor the nodule as recommended	39%
Document the results in the patient’s medical record	18%
Review report thoroughly	10%

**Table 7:** General suggestions based on brain MRI reports

Suggestion for a patient	Frequency
Follow-up with recommended appointments	100%
Follow-up with doctors	99%
Maintain a healthy lifestyle	97%
Monitor symptoms and report any changes to a healthcare provider	42%
Suggestion for a healthcare provider	Frequency
Communicate the findings clearly to the patient	100%
Schedule follow-up appointments	97%
Consider referral to a specialist	80%
Comprehensive treatment plan	53%
Evaluate the patient’s overall health	36%
Review report thoroughly	32%
Additional imaging	28%
Encourage a healthy lifestyle	17%

### Robustness of ChatGPT’s translations

It was found that ChatGPT’s translation was not uniquely confined to a certain radiology report format with different lengths of reorganized paragraphs and flexible choices of alternative words. Hence, it is necessary to investigate the randomness of ChatGPT’s responses. Ten translations of the same chest CT radiology report were collected and investigated. The original radiology report was split into 25 key information points and then evaluated for the correctness and completeness of each corresponding point in every translated report in a point-by-point fashion. The results of the chest CT radiology reports are shown in Table 8, where ‘Good’ means that information was clearly translated, ‘Missing’ indicates that an information point was completely lost in the translation, ‘Inaccurate’ stands for only partial information kept in the translated report, and ‘Incorrect’ indicates ChatGPT’s misinterpretation of the original radiology report. The overall ‘Good’ translation accounted for 55.2% of all the translated reports; 19.2%, 24.8%, and 0.8% of the information points were completely omitted, partially translated, and misinterpreted, respectively. Notably, for the translation of lung nodule findings, all 10 translations only mentioned the stable status of existing nodules compared with the previous screening and failed to provide detailed information, such as the precise position and size of each nodule. Therefore, all lung nodule findings were considered to be inaccurately translated. Although the observation of “no new nodules” was mentioned in the original report, only one translation reflected that point, and the other nine translations solely mentioned the stable status of existing nodules and omitted the statement that there were no new nodules during this screening. Only two instances of incorrect information occurred in the translation of patients’ smoking history. ChatGPT erroneously translated 30 packs per year as 30 years. ChatGPT sometimes neglected the minor problems mentioned in the original report. The lung finding of “mild emphysema with minor central bronchial wall thickening bilaterally” was only translated into mild emphysema in most translations, and the other minor finding of “normal caliber thoracic aorta with minor atherosclerotic change” was neglected in nine of the ten translations.

**Table 8:** Statistics regarding 10 repeated translations of a chest CT report

		Good	Missing	Inaccurate	Incorrect
Description	Lung CT screen without contrast	6	4	-	-
	Scanned on February 13, 2023	2	8	-	-
Indication	Lung cancer screening	10	-	-	-
	Patient smoked 30 or more packs per year	4	-	4	2
Comparison	February 11, 2022	2	8	-	-
Technic	Low dose axial CT, “as low as reasonably achievable” protocol	10	-	-	-
Findings					
Lung nodules	Lung nodule 1: nodule in right upper lobe, 4.9 mm x 3.4 mm, stable	-	-	10	-
	Lung nodule 2: pleura-based nodule in right middle lobe, 4.6 mm, stable	-	-	10	-
	Lung nodule 3: nonsolid round nodule in right lower lobe, 4.2 mm, stable	-	-	10	-
	Lung nodule 4: nonsolid subpleural round nodule in right lower lobe 4.6 mm, stable	-	-	10	-
	Lung nodule 5: subpleural nodule in right lower lobe, right lower lobe, 3 mm, stable	-	-	10	-
	No new nodules	1	9	-	-
Lung	Linear atelectasis and/or scarring in the right upper lobe, right middle lobe, lingula, and left lower lobe is mild	10	-	-	-
	Mild emphysema in the upper lung fields with minor central bronchial wall thickening bilaterally	2	-	8	-
Pleura	No pleural thickening or effusion	10	-	-	-
	No pneumothorax	10	-	-	-
Heart	Heart size normal	10	-	-	-
	No pericardial effusion	10	-	-	-
Coronary artery calcification	None	10	-	-	-
Mediastinum/ Hilum/Axillla	No adenopathy	8	2	-	-
Other	Normal caliber thoracic aorta with minor atherosclerotic change	1	9	-	-
Conclusion					
Overall Lung-RADS category	2-benign appearance or behavior	10	-	-	-
Based on lesion	ID multiple right-sided pulmonary nodules largest in the right upper lobe measuring 4.9 mm	2	8	-	-
Management recommendation	Continue annual screening with low dose CT in 12 months, February 2024	10	-	-	-
S findings	Minor sequela of COPD	10	-	-	-

### Optimized prompt for improved translation

It was found that ChatGPT tends to generate different responses for the same input, reflecting the uncertainty of the language model. Such randomness can compromise the quality of the translated results. One reason for ChatGPT’s varied responses was the ambiguity of the prompts. Instead of prompting ChatGPT to translate a radiology report into plain language, the initial prompts were optimized to be comprehensive and specific. The optimized prompts are as follows:

*Please help translate a radiology report into plain language in the following format:*

- *First paragraph introduces screening description including reason for screening, screening time, protocol, patient background, and comparison date;*
- *Second paragraph talks about specific findings: how many nodules detected, each lung nodule’s precise position and size, findings on lungs, heart, pleura, coronary artery calcification, mediastinum/hilum/axilla, and other findings. Please don’t leave out any information about findings;*
- *Third paragraph talks about conclusions, including overall lung-rads category, management recommendation and follow-up date, based on lesion;*
- *If there are incidental findings, please introduce in the fourth paragraph.*

With these prompts, 10 more ChatGPT plain-language translations of the radiology report were collected and subjected to the same statistical analyses as in the previous subsection. The results are summarized in Table 9. With the much clearer prompt, the overall quality of translation increased from 55.2% to 77.2%, and the measures on information that were completely omitted, partially translated, and misinterpreted were reduced to 9.2%, 13.6%, and 0%, respectively. A good example of using a detailed prompt is the translation of Lung nodule 1. In the experiment with a vague prompt, there was no translation which maintained the information in the original report. With a detailed prompt, eight out of ten translations presented the information on this nodule.

**Table 9:** Statistics regarding 10 repeated translations of a chest CT report with the optimized prompt

		Good	Missing	Inaccurate	Incorrect
Description	Lung CT screen without contrast	3	7	-	-
	Scanned on February 13, 2023	10	-	-	-
Indication	Lung cancer screening	10	-	-	-
	Patient who has smoked 30 or more packs per year	10	-	-	-
Comparison	February 11, 2022	10	-	-	-
Technic	Low dose axial CT, “as low as reasonably achievable” protocol	10	-	-	-
Findings					
Lung nodules	Lung nodule 1: nodule in right upper lobe, 4.9 mm x 3.4 mm, stable	8	-	2	-
	Lung nodule 2: pleura-based nodule in right middle lobe, 4.6 mm, stable	4	-	6	-
	Lung nodule 3: nonsolid round nodule in right lower lobe, 4.2 mm, stable	3	-	7	-
	Lung nodule 4: nonsolid subpleural round nodule in right lower lobe 4.6 mm, stable	3	-	7	-
	Lung nodule 5: subpleural nodule in right lower lobe, right lower lobe, 3 mm, stable	3	-	7	-
	No new nodules	4	6	-	-
Lung	Linear atelectasis and/or scarring in the right upper lobe, right middle lobe, lingula, and left lower lobe is mild	10	-	-	-
	Mild emphysema in the upper lung fields with minor central bronchial wall thickening bilaterally	7	-	3	-
Pleura	No pleural thickening or effusion	10	-	-	-
	No pneumothorax	8	2	-	-
Heart	Heart size normal	9	1	-	-
	No pericardial effusion	10	-	-	-
Coronary artery calcification	None	9	1	-	-
Mediastinum/ Hilum/Axillla	No adenopathy	9	1	-	-
Other	Normal caliber thoracic aorta with minor atherosclerotic change	8	2	-	-
Conclusion					
Overall Lung-RADS category	2-benign appearance or behavior	8	-	2	-
Based on lesion	ID multiple right-sided pulmonary nodules largest in the right upper lobe measuring 4.9 mm	7	3	-	-
Management recommendation	Continue annual screening with low dose CT in 12 months, February 2024	10	-	-	-
S findings	Minor sequela of COPD	10	-	-	-

### Different prompts on ChatGPT’s performance

The effect of engineering the prompts on ChatGPT’s performance was further investigated. Specifically, the first prompt was changed to the following formats:

- Please translate a radiology report into plain language for a patient only with high school education.
- Please translate a radiology report into plain language for a patient only with undergraduate education.
- Please translate a radiology report into plain language for a patient only with graduate education.
- Can you translate a radiology report into plain language that someone without medical training can easily understand?
- Your task is to translate a radiology report into plain language that is easy for the average person to understand. Your response should provide a clear and concise summary of the key findings in the report, using simple language that avoids medical jargon. Please note that your translation should accurately convey the information contained in the original report while making it accessible and understandable to a layperson. You may use analogies or examples to help explain complex concepts, but you should avoid oversimplifying or leaving out important details.

The first three prompts asked ChatGPT to translate radiology reports according to different educational levels. The fourth prompt was designed by ChatGPT based on the prompt “Please design the best prompt for you based on this prompt: Please translate a radiology report into plain language that is easy to understand.” The last prompt was designed using the website ‘promptperfect’ [15]. These five prompts are labeled as prompts 1-5, respectively.

**Fig. 1:**
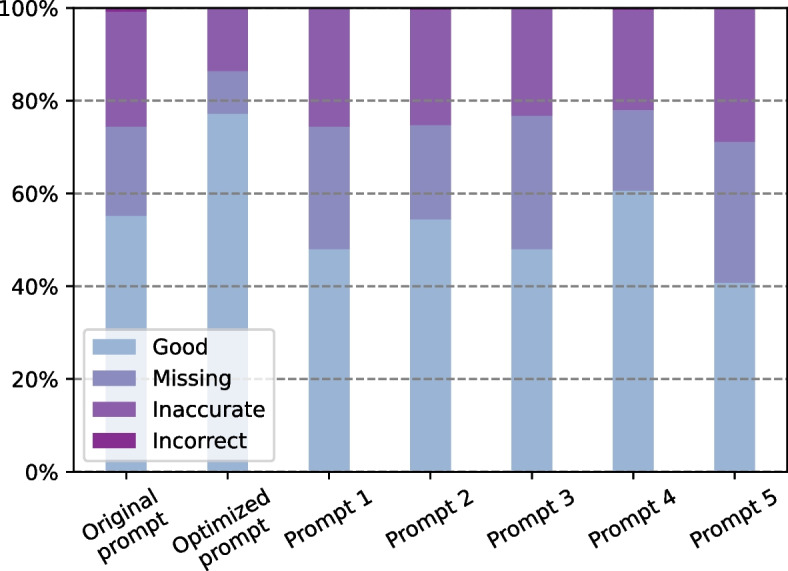
Effects of different prompts on ChatGPT’s translation performance

ChatGPT’s responses to these prompts were evaluated using the same method as that in the preceding subsection and were compared with the previous results from the original and the optimized prompts. The results are shown in Fig.  1. All of the five further-modified prompts were found to produce results similar to those of the original prompt and far worse than those of the optimized prompt. In terms of the five modified prompts, the fourth prompt designed by ChatGPT performed slightly better than the other four prompts, with a higher ‘Good’ rate and lower missing and inaccuracy rates. However, the fourth prompt continued to perform significantly worse than the optimized prompt described in the preceding subsection.

### ChatGPT’s ensemble learning results

In the above subsections, ChatGPT was asked to generate multiple translated reports for the same prompts and radiology reports. In this study, ChatGPT’s performance was further investigated via ensemble learning. In each case, five translated reports were randomly selected and input into ChatGPT for information integration. ChatGPT was asked to combine all the results to create a single report. Statistics of 10 ensemble learning results are presented in Table 10. In general, ChatGPT could not generate significantly better results using ensemble learning. Although ChatGPT performed better when combining the results obtained from the original prompt, with a higher ‘Good’ rate and lower missing, inaccurate, and incorrect rates, such an improvement is not significant compared with the improvement obtained by replacing the original prompt with the optimized prompt. In this experiment, the incremental increase in the ‘Good’ rate was attributed to reporting more scan details, such as pointing out that scans were without contrast, and additional details on the patient’s smoking history. Upon integrating the results obtained with the optimized prompt, the ‘Good’ rate declined, while the missing and accurate rates increased. This inferior performance mainly resulted from the over-simplification of lung nodule findings and the overlooking of minor findings, such as normal caliber thoracic aorta with minor atherosclerotic changes.

**Table 10:** Percentage change of ensemble learning vs non-ensemble results

	Good	Missing	Inaccurate	Incorrect
Original prompt	6.4%	-0.8%	-4.8%	-0.8%
Optimized prompt	-4.4%	1.2%	3.2%	0%

### Comparison with GPT-4

On March 14, 2023, OpenAI launched its new large language model, GPT-4, with an impressive performance on multimodal tasks [16]. The performance of GPT-4 was subsequently investigated in the radiology report translation task and was compared with that of ChatGPT. The experiments were conducted using the original and optimized prompts with the same methodology as that used in the ChatGPT experiment. According to Table 11, GPT-4 significantly improves the quality of the translated reports, with higher ‘Good’ rates and lower missing and inaccurate rates using both the original and optimized prompts; impressively, GPT-4’s performance with the original prompt was competitive with that of ChatGPT using the optimized prompt, and GPT-4 with the optimized prompt achieved a ‘Good’ rate of almost 100%.

Similar to ChatGPT, GPT-4 exhibits some randomness. In the experiment using the optimized prompt, a translation failed to follow the provided format. According to the required format, incidental findings should be listed in the fourth paragraph, but GPT-4 showed the incidental findings of chronic obstructive pulmonary disease in the third paragraph along with conclusions.

**Table 11:** Comparison of GPT-4 and ChatGPT on the radiology report plain language translation task

		Good	Missing	Inaccurate	Incorrect
ChatGPT	Original prompt	55.2%	19.2%	24.8%	0.8%
	Optimized prompt	77.2%	9.2%	13.6%	0%
GPT-4	Original prompt	73.6%	8.0%	18.4%	0%
	Optimized prompt	96.8%	1.6%	1.6%	0%

## Discussion

ChatGPT, as the first publicly available convincing step toward artificial general intelligence, has already demonstrated an excellent ability for organizing words and sentences. ChatGPT can be used for multiple purposes, such as writing news, telling stories, and language translations. In this study, its potential for translating radiology reports into plain language and making suggestions based on these reports was evaluated. According to the results, ChatGPT provides at least three advantages in radiology report translations: conciseness, clarity, and comprehensiveness. In terms of conciseness, ChatGPT deletes redundant words from the original report and summarizes multiple findings in a single sentence. Regarding clarity, commonly used words are adopted by ChatGPT to replace complicated medical terminology so that patients with different educational backgrounds can easily digest the information. In terms of comprehensiveness, ChatGPT has a strong ability to understand the original radiology report and to integrate information from different sections of the original report into easily understandable sentences.

Our experiment also revealed the uncertainty in ChatGPT’s responses. Given the same prompt for the same radiology report, ChatGPT generates distinctive responses each time, which results in a variety of translated reports. Such random results are partially inherent to the language model and partially attributed to the ambiguity of the prompts. The original prompt only gave ChatGPT a generic instruction to translate a radiology report into plain language, and there were no specific instructions on which information was important and should be kept. As a result, ChatGPT tended to generate oversimplified translations that excluded important information. The results suggest a weakness in the current version of ChatGPT: it does not know which information is important and should be included in a radiology report. The experiment with an optimized prompt with detailed instructions on which information should be retained demonstrates that ChatGPT can generate improved results with clearer and more specific instructions. Meanwhile, ChatGPT exhibits semantic robustness when there is no significant difference between different prompts. According to Fig. 1 and Table 10, when there are no clear instructions on the information to retain, ChatGPT performance is similar to that of multiple semantically similar prompts.

Another notable finding is that ChatGPT does not have a built-in template for the generated report translations. Radiology reports usually follow a fixed template; therefore, reports prepared by different radiologists are presented in a consistent style. Such a consistent template significantly improves the efficiency of radiology report generation and saves time for healthcare providers in digesting radiology reports. The results show that ChatGPT tends to generate results in various formats when the prompt has no formatting instructions. In some cases, ChatGPT produces a single-paragraph translation combining all findings and conclusions. Compared with translated reports that have multiple paragraphs and present information on screening descriptions, findings, and conclusions in different paragraphs, a single-paragraph translated report may be more difficult for patients to read. Designing a prompt with clear instructions on the format of the translated reports can help ChatGPT generate translations with a consistent structure for better readability. For example, the number of paragraphs and words can be added to the prompt to specify the format and length of the translation.

According to the evaluation results of the consulted radiologists, ChatGPT’s translated results contained little missing information or misinterpretations, alleviating concerns about the reliability of ChatGPT’s translation results. Currently, large language models are rapidly developing, and new models are being frequently released;. for instance, GPT-4 was launched on March 14, 2023, with the ability to handle multimodal data such as text and images. It performs better on multiple tasks such as a uniform bar exam than its predecessor GPT-3.5 [17]. The results on GPT-4 also demonstrate a significant improvement compared with ChatGPT. In the future, it will be desirable to utilize large language models for clinical applications.

Despite the potential of ChatGPT in radiology report translation, concerns remain regarding its deployment in clinical practice. The first concern is that ChatGPT’s report translation still lacks completeness and may leave out some key points. Based on the results, using an optimized prompt can improve completeness; however, the current results are not perfect. Another concern is the inconsistency or uncertainty of ChatGPT’s responses. ChatGPT may provide inconsistent translations and present information in variable formats with potential oversimplification or information loss for the same radiology report with the same prompt.

In terms of using artificial intelligence in the healthcare domain, large language models such as ChatGPT have demonstrated their potential. This study is a good example demonstrating that radiology reports can be efficiently and effectively translated into plain language, even automatically with useful suggestions, without direct involvement of human experts. In the future, ChatGPT-type systems will surely be extensively used in healthcare to provide great assistance, such as generating full radiology reports directly from medical images, analyzing treatment options and plans, guiding patients’ daily lives by considering all their medical data, and providing psychological counseling as needed.

Clearly, ChatGPT and its products will greatly impact the way medical information is formulated, queried, and shared across patients and healthcare providers. The evidence needed to demonstrate to regulators that such algorithms are safe and effective will depend on their intended use as well as the risks and benefits associated with the intended use. Tools that support communication between healthcare providers and patients are more likely to be accepted as safe than tools that have a more direct impact on patient diagnosis and treatment planning. Further developments of these products and additional evaluations of their performance characteristics for purposes of regulatory review and user adoption would be welcome.

## Conclusion

The feasibility and utility of ChatGPT in low-level clinical applications were analyzed, specifically in the translation of radiology reports into plain language to make recommendations to a patient or a healthcare provider. Experiments were conducted to evaluate ChatGPT’s performance in this particular clinical task. Based on the evaluation results, ChatGPT’s translations had an overall score of 4.268 on a five-point system (5 for best and 1 for worst), with an average of 0.097 places of missing information and an average of 0.065 places of incorrect information per translation. Regarding the uncertainty of ChatGPT’s responses, it was found that ChatGPT’s plain language translation tends to oversimplify or overlook some key points, with only 55.2% of the key points completely translated when using an ambiguous prompt. Such uncertainty can be reduced, thereby retaining 77.2% of the full information by replacing the vague prompt with an optimized prompt. ChatGPT was further compared with GPT-4, and it was found that GPT-4 significantly improved the quality of the translated reports. This study demonstrated that advanced large-language models such as ChatGPT and GPT-4 are promising new tools in clinical applications, and that a project on the translation of radiology reports into plain language would be an excellent preliminary application of this technology.

## Data Availability

For accessing radiology reports used in this paper, please contact the corresponding author Christopher T. Whitlow.

## Abbreviations

NLP: Natural language processing
GPT: Generative pre-trained transformer
RLHF: Reinforcement learning from human feedback
CT: Computed tomography
MRI: Magnetic resonance imaging

## References

[1] ChatGPT sets record for fastest-growing user base-analyst note. https://www.marketscreener.com/news/latest/ChatGPT-sets-record-for-fastestgrowing-user-base-analyst-note--42873811/. Accessed 20 Feb 2023

[2] ChatGPT reaches 100 million users two months after launch. https://www.theguardian.com/technology/2023/feb/02/chatgpt-100-million-usersopen-ai-fastest-growing-app. Accessed 20 Feb 2023

[3] Devlin J, Chang MW, Lee K, Toutanova K (2019) BERT: Pre-training of deep bidirectional transformers for language understanding. In: Proceedings of the 2019 conference of the North American chapter of the association for computational linguistics: human language technologies, volume 1 (Long and Short Papers), Association for Computational Linguistics, Minneapolis, 2-7 June 2019

[4] Yang ZL, Dai ZH, Yang YM, Carbonell J, Salakhutdinov R, Le QV (2019) XLNet: Generalized autoregressive pretraining for language understanding. In: Proceedings of the 33rd international conference on neural information processing systems, Curran Associates Inc., Vancouver, 8 December 2019

[5] Radford A, Narasimhan K, Salimans T, Sutskever I (2018) Improving language understanding by generative pre-training

[6] Ouyang L, Wu J, Jiang X, Almeida D, Wainwright CL, Mishkin P et al. (2022) Training language models to follow instructions with human feedback. arXiv preprint arXiv:2203.02155

[7] Wang S, Scells H, Koopman B, Zuccon G (2023) Can ChatGPT write a good Boolean query for systematic review literature search? arXiv preprint arXiv:2302.03495. 10.1145/3539813.3545143

[8] Kung TH, Cheatham M, Medenilla A, Sillos C, De Leon L, Elepaño C et al. (2023) Performance of ChatGPT on USMLE: Potential for AI-assisted medical education using large language models. PLoS Digit Health 2(2):0000198. 10.1371/journal.pdig.0000198 10.1371/journal.pdig.0000198 PMC9931230 36812645

[9] Liebrenz M, Schleifer R, Buadze A, Bhugra D, Smith A (2023) Generating scholarly content with ChatGPT: ethical challenges for medical publishing. Lancet Digit Health 5(3):E105–E106. 10.1016/S2589-7500(23)00019-5 10.1016/S2589-7500(23)00019-5 36754725

[10] Patel SB, Lam K (2023) ChatGPT: the future of discharge summaries? Lancet Digit Health 5(3):E107–E108. 10.1016/S2589-7500(23)00021-3 10.1016/S2589-7500(23)00021-3 36754724

[11] Biswas S (2023) ChatGPT and the future of medical writing. Radiology 307(2):e223312. 10.1148/radiol.223312 10.1148/radiol.223312 36728748

[12] Jeblick K, Schachtner B, Dexl J, Mittermeier A, Stüber AT, Topalis J et al. (2022) ChatGPT makes medicine easy to swallow: an exploratory case study on simplified radiology reports. arXiv preprint arXiv:2212.14882 10.1007/s00330-023-10213-1 PMC11126432 37794249

[13] Rao A, Kim J, Kamineni M, Pang M, Lie W, Succi MD (2023) Evaluating ChatGPT as an adjunct for radiologic decision-making. medRxiv, 2023-02. 10.1101/2023.02.02.23285399 10.1016/j.jacr.2023.05.003 PMC10733745 37356806

[14] Sarraju A, Bruemmer D, Van Iterson E, Cho L, Rodriguez F, Laffin L (2023) Appropriateness of cardiovascular disease prevention recommendations obtained from a popular online chat-based artificial intelligence model. JAMA 329(10):842–844. 10.1001/jama.2023.1044 10.1001/jama.2023.1044 PMC10015303 36735264

[15] PromptPerfect: elevate your prompts to perfection. https://promptperfect.jina.ai/. Accessed 20 Feb 2023

[16] OpenAI: GPT-4 technique report (2023) https://cdn.openai.com/papers/gpt-4.pdf. Accessed 14 Mar 2023

[17] GPT-4. https://openai.com/research/gpt-4. Accessed 14 Mar 2023

